# Effects of *Aphloia theiformis* on key enzymes related to diabetes mellitus

**DOI:** 10.1080/13880209.2016.1277765

**Published:** 2017-02-01

**Authors:** Marie Carene Nancy Picot, Mohamad Fawzi Mahomoodally

**Affiliations:** Department of Health Sciences, Faculty of Science, University of Mauritius, Réduit, Mauritius

**Keywords:** α-Amylase, α-glucosidase, pancreatic lipase, Mauritius

## Abstract

**Context:***Aphloia theiformis* (Vahl.) Benn. (Flacourtiaceae) (AT) is traditionally used for the management of diabetes mellitus (DM), but there is no scientific data regarding activity against enzymes linked to this condition.

**Objective:** To evaluate the kinetics of AT on key enzymes inhibition related to DM, and establish the antioxidant profile of AT.

**Materials and methods:** Dried powdered AT leaves were used to prepare crude methanol extract (70% v/v) (CME). Kinetics of CME (5000 to 156.25 μg/mL) on α-amylase, α-glucosidase, and lipase inhibition were studied. CME was partitioned using solvents of increasing polarity and kinetics of enzyme inhibition of each fraction (1000–31.25 μg/mL) was evaluated. Potent fractions were combined to assess any synergistic effect. Total phenol, flavonoid, tannin, anthocyanin contents, and antioxidant capacity of AT was evaluated using standard spectrophotometric methods.

**Results:** CME, ethyl acetate, and *n*-butanol fractions showed potent inhibitory activities against the enzymes with IC_50_ ranging from 22.94–939.97 μg/mL. Significant (*p* < 0.05) reduction in IC_50_ (15.72 and 157.03 μg/mL against α-amylase and lipase, respectively) was observed when ethyl acetate and *n*-butanol fractions were combined; showing synergism. The extracts showed noncompetitive inhibition against α-amylase and α-glucosidase. Ethyl acetate, *n*-butanol fractions, and CME showed highest antioxidant capacities (0.44–1.41 μg GAE/mg sample), and phenol content (211.74-675.53 μg GAE/mg sample).

**Conclusion:** This study supports the use of AT in the management of DM and provides the rationale for bioactivity guided isolation and characterization of compounds from the ethyl acetate and *n*-butanol fractions.

## Introduction

*Aphloia theiformis* (Vahl.) Benn. (Flacourtiaceae) (AT) is indigenous to Madagascar, Comoros Island, Mascarenes Islands, Seychelles Archipelago and tropical Africa (Antoine et al. 1993; Gurib-Fakim & Brendler 2004). Aphloia’ comes from the Greek epithet ‘a’ meaning ‘without’ and ‘phloios’ meaning ‘bark, skin, flower’ (Gurib-Fakim 2002). ‘Theiformis’ comes from the Latin epithet ‘thea’ meaning ‘tea’ and ‘formis’ meaning ‘form’ (Gurib-Fakim & Brendler 2004). Some common vernacular names include Fandamane and Bois goyave/gouyave in Mauritius, Change écorce/Bois change écorce and Gouyavier marron/Gouyave marron in Réunion Island, Bois d’anémone in Rodrigues, Bwa merl/Bois merle in Seychelles, Voafotsy, Fandramanana, and Maramanana in Madagascar (Antoine et al. 1993; Gurib-Fakim 2003; Gurib-Fakim & Brendler 2004; Danthu et al. 2010).

AT is presented as a treelet reaching 15 m tall with a truck measuring 30 cm in diameter. The trunk is black to blackish brown, deciduous in patches; the underlying bark is smooth and pale brown. The young branches are reddish and striated. Mature leaves are variable in shape with narrow to broadly elliptic or narrowly oboval to oboval-elliptic blade. The leaf margin is denticulate to glandular-dentate. Flowers are auxiliary, solitary or in bunch with white to pale yellow sepals. The fruits are sub-globular to ovoid-pyriform and are white at maturity (Antoine et al. 1993; Gurib-Fakim & Brendler 2004).

Ethnopharmacological data has revealed that AT has been extensively used alone or in combination with other medicinal plants for the management of various diseases including dysentery, fever, as a diuretic, rheumatism, ulcers, jaundice, gastrointestinal infections, skin infections (Gurib-Fakim & Brendler 2004), cataract, decrease cholesterol level, diabetes mellitus (Mootoosamy & Mahomoodally 2014), wound healing and anti-pyretic (Danthu et al. 2010). It has been previously reported that AT leaves are rich in xanthones which possess ‘anti-aging’ and photo-protective properties (Danthu et al. 2010). Another study conducted by Gopalsamy et al. (1988) showed the presence of saponins in AT leaves, namely, tormentic acid ester glucoside, 23-hydroxytormentic acid ester glucoside, and 6-β-hydroxytormentic acid ester glucoside. The use of AT leaves for the treatment of inflammatory and immune dysfunctions was investigated by Hsoidrou et al. (2014). Phenol fraction of AT leaves produced immuno-stimulatory effects on monocytes and granulocytes and showed anti-inflammatory properties in carrageenan-induced paw oedema in rats. Crude methanol extract of AT leaves showed antimicrobial potential against *Staphylococcus aureus, Salmonella enteritis, Pseudomonas aeruginosa, Enterobacter cloacae, Bacillus subtilis, Sclerotinia sclerotium*, and *Candida albicans* using the disc diffusion technique (Rangasamy et al. 2007).

Interestingly, an ethnopharmacological study conducted by Mootoosamy and Mahomoodally (2014) reported the use of AT in the management of diabetes mellitus and obesity. To our knowledge, there have been no attempts to evaluate the possible inhibitory activity of AT on key enzymes linked to these closely associated conditions. The main aim of the present study is to evaluate the possible inhibitory action of AT on α-amylase, α-glucosidase, and pancreatic lipase using *in vitro* techniques.

## Materials and methods

### Plant material

The leaves of AT (bar-code number: MAU 26544) used in this study were collected on the 5th of May 2015 from Montvert Nature Reserve situated on the upper regions of Mauritius and authenticated by Mr. K. Pynee, Senior Technical Assistant/Botanist of the Mauritius Herbarium Agricultural Services.

### Apparatus

Dried leaves were ground using a Pacific mixer grinder (India). For concentration of filtrates *in vacuo*, a rotary evaporator (Stuart rotavap and digital water bath manufactured by Bibby Scientific Ltd, UK) was used. Spectrophotometric determinations were carried out using a Thermo Scientific Genesys 10S UV-Vis spectrophotometer. Micro-plates were read using ELISA micro-plate reader (Labsystems Multiskan, MS 352, Finland).

### Extraction

The leaves (800 g) were washed under running tap water to remove soil and debris and shade dried. The dried plant material (415 g) was then pulverized. Dried plant material (250 g) was exhaustively extracted with 2 L of methanol at 70% (v/v). The extract was filtered and the filtrate was concentrated to about ¼ of its original volume under reduced pressure. About ¼ of the concentrated filtrate was afforded into a paste and the remainder was used for solvent partitioning. The aqueous crude extract was prepared following decoction method. Briefly, 50 g of dried powdered material was boiled into 200 mL distilled water for 30 min. The filtrate obtained was concentrated under reduced pressure. The crude methanol and aqueous extracts were subjected to enzymatic, antioxidant, and phytochemicals evaluations as described below.

### Solvent partitioning

The crude methanol extract was suspended in distilled water and successively partitioned using four solvents of increasing polarity, namely hexane, dichloro-methane, ethyl acetate, and *n*-butanol. The resulting solvent fractions were concentrated *in vacuo* (Aderogba et al. 2013; Ajileye et al. 2015). Each fraction was subjected to enzymatic, antioxidant, and phytochemicals evaluations as described below.

### α-Amylase inhibition assay

The α-amylase (Sigma-Aldrich, UK) inhibitory activity was measured as described by Mahomoodally et al. (2012) and Kotowaroo et al. (2006). Briefly, 100 μL of sample solution (initial concentrations used ranged from 5000 to 156.25 μg/mL for crude extracts and 1000 to 31.25 μg/mL for fractions) was pre-mixed with 100 μL of α-amylase solution (13 U/mL in 0.1 M sodium acetate buffer pH 7.2) and incubated at 37 °C for 15 min. After pre-incubation, 3 mL of soluble starch solution (1% w/v) was added to the enzyme-sample mixture to initiate the reaction followed by 2 mL sodium acetate buffer (0.1 M, pH 7.2). An aliquot from the reaction mixture was discharged into 10 mL iodine solution (0.254 g iodine and 4 g potassium iodide was made up to 1 L using distilled water) and the absorbance was measured at 565 nm. The α-amylase inhibitory activity was expressed as percentage (%) inhibition as follows: [1−(Abs_control_−Abs_sample_)/Abs_control_] × 100.

The concentration of sample required to inhibit α-amylase activity by 50% (IC_50_) under assay conditions was calculated from the percentage inhibition values. For kinetic analyses, the enzyme and sample were incubated with increasing concentrations of starch. The kinetics of inhibition was analyzed using Line-weaver Burke plots.

### α-Glucosidase inhibition assay

The α-glucosidase (Sigma-Aldrich, UK) inhibitory activity was measured as described by Bachhawat et al. (2011) and Mayur et al. (2010) with slight modifications. A volume of 20 μL of sample solution (initial concentrations used ranged from 5000 to 156.25 μg/mL for crude extracts and 1000 to 31.25 μg/mL for fractions) was pre-mixed with 10 μL of α-glucosidase solution (1 U/mL in 0.1 M phosphate buffer pH 6.9) and incubated at 37 °C for 15 min. After pre-incubation, 20 μL of *p*-nitrophenol-α-d-glucopyranoside (PNPG) (1 mM) was added to start the reaction. The reaction mixture was made up to 100 μL using 0.1 M phosphate buffer pH 6.9. The reaction was carried out at 37 °C for 30 min and terminated by adding of 50 μL sodium carbonate (0.1 M). The absorbance was read at 405 nm. The % inhibition and IC_50_ were calculated as described above. For kinetic analyses, the enzyme and sample were incubated with increasing concentration of PNPG solution. The kinetics of inhibition was calculated using Line-weaver Burke plots.

### Pancreatic lipase inhibition assay

The pancreatic lipase (Sigma-Aldrich, UK) inhibitory activity was determined as described by Bustanji et al. (2010) with modifications. A volume of 50 μL pancreatic lipase solution (1 mg/mL in 2.5 mM tris-hydrochloride buffer pH 7.4 with 0.125 mM sodium chloride) was pre-mixed with 100 μL sample solution (initial concentrations used ranged from 5000 to 156.25 μg/mL for crude extracts and 1000 to 31.25 μg/mL for fractions) and incubated at 37 °C for 15 min. Following pre-incubation, 100 μL *p*-nitrophenyl butyrate (PNPB) (25 mM) was added to the enzyme-sample mixture and the volume was made up to 300 μL using tris-hydrochloride buffer. The reaction mixture was incubated at 37 °C for 60 min and the amount of *p*-nitro-phenol released was measured at 405 nm. The % inhibition and IC_50_ were calculated as previously described. The kinetics of inhibition was calculated by increasing the concentration of PNPB and kinetic parameters were calculated from Line-weaver Burke plots.

### Synergistic effect of the most potent fractions

In order to determine the possible synergistic effect of the most potent fractions against α-amylase, α-glucosidase, and pancreatic lipase, equal volumes of the two most potent fractions with the same concentration were mixed (Wang et al. 2010). The percentage inhibition was calculated from a concentration gradient and was used to determine the IC_50_ value. The reactions were carried out as previously described and the IC_50_ values were calculated. Synergism was considered when the IC_50_ values of the mixture were significantly different from the IC_50_ values of the fractions alone.

### Antioxidant assays

#### Phospho-molybdenum assay

The antioxidant capacity of the samples was assessed based on the reduction of molybdenum (VI) to molybdenum (V) which produced a green phospho-molybdenum (V) complex under acidic conditions (Chaouche et al. 2014). Briefly, 100 μL of the sample was mixed with 1 mL of reagent solution containing 0.6 M sulfuric acid, 28 mM sodium phosphate and 4 mM ammonium molybdate. The reaction mixture was incubated at 95 °C for 90 min. After the incubation period the reaction mixture was allowed to cool and the absorbance was measured at 695 nm. The antioxidant capacity was expressed as μg gallic acid equivalent (GAE)/mg crude extract/fraction using gallic acid calibration curve.

#### Ferric reducing antioxidant power (FRAP) assay

The ferric-reducing antioxidant power (FRAP) of the samples was determined according to the modified method of Benzie and Strain (1996). The sample was mixed with 2850 μL FRAP solution containing 25 mL acetate buffer, 2.5 mL 2-4-6 tripyridyl-s-triazine (10 mM) in hydrochloric acid (40 mM) and 2.5 mL hydrated ferric chloride solution (20 mM). The reaction was allowed for 30 min in the dark and absorbance was read at 593 nm. Data were expressed as mM Trolox Equivalent (TE)/mg crude extract/fraction using Trolox calibration curve.

#### 2,2-Diphenyl-1-picrylhydrazyl hydrate (DPPH) free radical scavenging assay

The free radical scavenging activity was measured using the DPPH assay as described by Umamaheswari et al. (2008). Briefly, 200 μL freshly prepared DPPH solution (100 μM in methanol) and 100 μL of sample in methanol was incubated at 37 °C for 30 min. After incubation, the absorbance was measured at 517 nm. The inhibition of DPPH was calculated and concentration of sample required to cause half reduction in DPPH radical absorbance (IC_50_) was calculated.

#### β-Carotene linoleic acid assay

The antioxidant activity of the sample was evaluated as described by Gholivand et al. (2014) with some modifications. β-Carotene solution (210 mL, 0.5 mg/mL in chloroform), 5 μL linoleic acid solution and 42 μL Tween 20 solution were pipetted into a round bottom flask. The chloroform was removed by rotary vacuum evaporator at 40 °C, 10 mL deionized water was added to the residue and the mixture was vigorously shaken to form an emulsion. Sample in methanol (50 mL) was added to 200 μL of the emulsion and incubated for 2 h at 50 °C. After incubation period, absorbance was measured at 450 nm. The bleaching inhibition and IC_50_ were calculated.

### Phytochemical determination

#### Determination of total phenol content

Total phenol content (TPC) was determined according to the modified Folin Ciocalteau assay as described by Nickavar and Esbati (2012). The reaction mixture containing 2500 μL of a 10-fold diluted Folin-Ciocalteau reagent solution, 500 μL sample and 2000 μL sodium carbonate (7.5%) was allowed to react for 30 min. The TPC of the samples was then spectrophotometrically determined at 760 nm. The results obtained were expressed as μg GAE/mg crude extract/fraction using gallic acid standard curve.

#### Determination of total flavonoid content

Total flavonoid content (TFC) was determined following the aluminum chloride colorimetric method as described by Amaeze et al. (2011). The reaction mixture consisted of 2 mL sample and 2 mL aluminum chloride solution (2%). The mixture was allowed to react for 30 min and the absorbance was read at 420 nm. Results were expressed as μg rutin equivalent (RE)/mg crude extract/fraction using rutin calibration curve.

#### Determination of total anthocyanin content

Total anthocyanin content (TAC) was determined based on the pH differential method (Sutharut & Sudarat 2012). Briefly, 1 mL of plant extract was transferred into 10 mL volumetric flask and the volume was adjusted with buffer pH 1.0 and pH 4.5. The mixtures were allowed to equilibrate for 15 min. The absorbance of each dilution was spectrophotometrically determined at 510 and 700 nm. The absorbance of diluted samples was determined using the following equation: A = (A_510_−A_700_)_pH1.0_−(A_510_−A_700_)_pH4.5_

The monomeric anthocyanin pigment concentration in the original sample was calculated according to the following equation:

$$\mathrm{Anthocyanin}\;\mathrm{content}\;\left(\mathrm{mg}/\mathrm{mL}\right)=\frac{A\times \mathrm{MW}\times \mathrm{DF}\times 1000}{\left(ɛ\times 1\right)}$$

where MW is the molecular weight of cyanidin-3-glucoside (484.5), DF the dilution factor, and ɛ the molar extinction coefficient (26,900).

#### Determination of total tannin content

Total tannin content (TTC) of the samples was measured using the vanillin-hydrochloride method as described by Mak et al. (2013). Briefly, 1 mL of the plant extract was mixed with 5 mL of reagent mixture (4% vanillin in methanol and 8% hydrochloric acid in methanol in the ratio of 1:1). The color formed after 20 min was spectrophotometrically determined at 500 nm. Results were expressed as μg catechin equivalent (CE)/mg crude extract/fraction using catechin calibration curve.

### Statistical analysis

Results were expressed as mean ± standard deviation of three independent determinations. Difference between the samples was determined using one way analysis of variance (ANOVA) followed by Tukey post-test with statistical significance considered as *p <* 0.05.

## Results

The IC_50_ values of the crude extracts and fractions of AT on α-amylase, α-glucosidase, and pancreatic lipase are presented in Table 1. The crude methanol extract of AT was an active inhibitor of α-amylase, α-glucosidase and pancreatic lipase as compared to the crude aqueous extract. Following fractionation of the crude methanol extract, it was observed that the ethyl acetate and *n*-butanol fractions were potent inhibitors of α-amylase, α-glucosidase, and pancreatic lipase. The IC_50_ value of *n*-butanol fraction of AT (22.94 μg/mL) for the inhibition of α-amylase was significantly (*p* < 0.05) lower than the control, acarbose (38.45 μg/mL). Ethyl acetate fraction of AT (43.57 μg/mL) also inhibited α-amylase, but showed an IC_50_ value significantly (*p* < 0.05) higher than acarbose. Both ethyl acetate and *n*-butanol fractions (45.58 and 23.44 μg/mL, respectively) showed IC_50_ values significantly (*p* < 0.05) lower than the control acarbose (749.48 μg/mL) for the inhibition of α-glucosidase. Ethyl acetate and *n*-butanol fractions inhibition of α-amylase, α-glucosidase, and pancreatic lipase were significantly (*p* < 0.05) different from controls (Table 1).

**Table 1.** IC_50_ values of crude methanol and aqueous extracts and fractions of AT against enzymes relevant to diabetes and related complications.

	Crude extract (μg/mL)		Fractions (μg/mL)					
Enzyme	Methanol	Aqueous	Hexane	DCM	Ethyl acetate	*n*-Butanol	Water	Controls
α-amylase	67.71 ± 1.21^a^	–	–	–	43.59 ± 1.59^d^	22.94 ± 0.40^b^	–	38.45 ± 1.36*^c^
α-glucosidase	55.20 ± 1.64^a^	77.63 ± 1.67^b^	–	–	45.58 ± 0.89^a^	23.44 ± 0.08^c^		749.48 ± 1.726*^d^
Pancreatic lipase	939.97 ± 49.34^b^	–	–	–	202.31 ± 23.91^c^	39.32 ± 1.32^a^	279.31 ± 6.45^d^	41.61 ± 6.08‡^a^

Values are expressed as mean ± standard deviation of triplicate determinations. Values in the same row having different superscripts differ significantly (*p* < 0.05) compared to control. – Low activity observed. *: acarbose, ‡: Orlistat^®^.

Table 2 shows the inhibitory potential of a mixture of ethyl acetate and *n*-butanol fractions against α-amylase, α-glucosidase, and pancreatic lipase. Synergism was observed by comparing the IC_50_ values of the mixture with that of individual fractions. A significant improvement was observed against α-amylase and pancreatic lipase since the IC_50_ values of the fractions were significantly (*p* < 0.05) different from the IC_50_ of the ethyl acetate/*n*-butanol mixture.

**Table 2.** Possible synergistic effect of ethyl acetate and butanol fractions on α-amylase, α-glucosidase and pancreatic lipase.

Sample	α-Amylase (IC_50_ μg/mL)	Interaction	α-Glucosidase (IC_50_ μg/mL)	Interaction	Pancreatic lipase (IC_50_ μg/mL)	Interaction
Ethyl acetate	43.59 ± 1.59^a^	Synergistic	45.58 ± 0.89^a^	None	202.31 ± 23.91^a^	Synergistic
*n*-butanol	22.94 ± 0.40^b^		23.44 ± 0.08^b^		39.32 ± 1.32^b^	
Ethyl acetate/*n*-butanol mixture	15.72 ± 0.73^c^		46.32 ± 0.60^a^		157.03 ± 4.73^c^	

Values are expressed as mean ± standard deviation of triplicate determinations. Values in the same column having different superscripts differ significantly (*p* < 0.05) from the ethyl acetate/*n*-butanol mixture.

**Figure 1.**
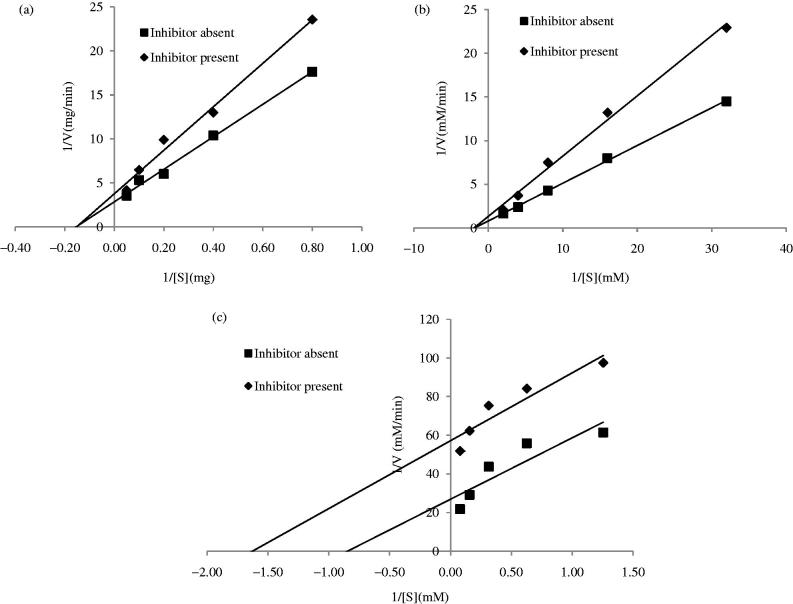
Double-reciprocal Lineweaver Burk plots of crude methanol extract of AT on (a) α-amylase, (b) α-glucosidase, and (c) pancreatic lipase.

The mode of inhibition of α-amylase, α-glucosidase, and pancreatic lipase by the most active crude extract, i.e. the methanol crude extract, was investigated as depicted in Figure 1. Crude methanol extract of AT displayed a pure noncompetitive inhibition against α-amylase and α-glucosidase as shown in Figure 1(a,b). As shown in Figure 1(c), the crude methanol extract of AT uncompetitively inhibited pancreatic lipase.

**Figure 2.**
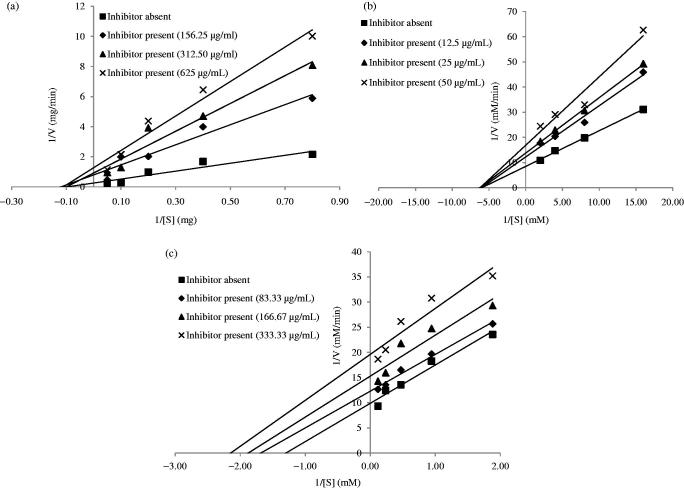
Double-reciprocal Lineweaver Burk plots of the different concentrations of ethyl acetate fraction of AT on (a) α-amylase, (b) α-glucosidase, and (c) pancreatic lipase.

**Figure 3.**
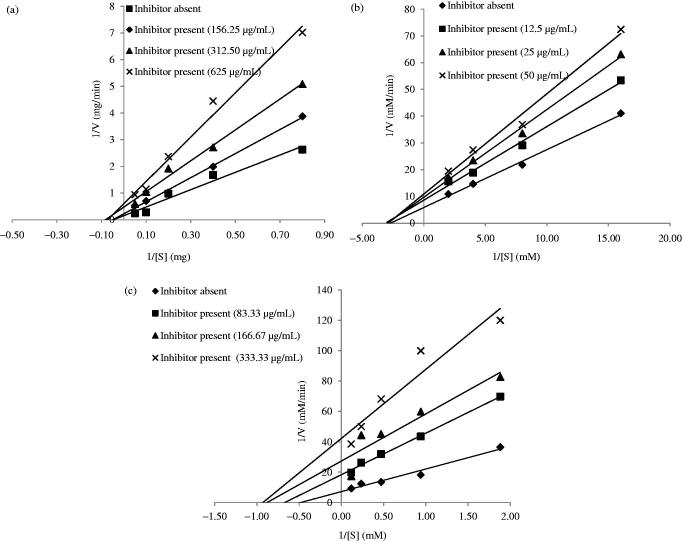
Double-reciprocal Lineweaver Burk plots of the different concentrations of *n*-butanol fraction of AT on (a) α-amylase, (b) α-glucosidase, and (c) pancreatic lipase.

Since, the ethyl acetate and *n*-butanol fractions of AT were the most potent fractions, their patterns of inhibition on the enzymes were investigated by varying the concentration of the fractions (Figures 2 and 3, respectively). From Figure 2, it was observed that the ethyl acetate fraction showed a noncompetitive mode of inhibition against α-amylase and α-glucosidase (Figure 2(a,b), respectively) and uncompetitive mode of inhibition against pancreatic lipase (Figure 2(c)). Similarly, *n*-butanol fraction noncompetitively inhibited α-amylase, α-glucosidase, and uncompetitively inhibited lipase as depicted in Figure 3(a–c).

Table 3 summarizes the antioxidant potential of crude extracts and fractions of AT. It was observed that the crude methanol extract showed higher antioxidant capabilities as compared to the crude aqueous extract. The antioxidant capacity of the crude methanol extract (0.44 μg GAE/mg crude extract) was significantly (*p* < 0.05) higher than the crude aqueous extract (0.17 μg GAE/mg crude extract). A similar trend was observed for the FRAP assay (1625.63 μg TE/mg crude methanol extract and 580.23 μg TE/mg crude aqueous extract). For the DPPH and β-carotene assays, low IC_50_ values depicted higher activity. The crude methanol extract of AT showed lowest IC_50_ for both assays. Following fractionation of the crude methanol extract, it was observed that the ethyl acetate and *n*-butanol fractions exhibited highest antioxidant capacities as shown in Table 3. The ethyl acetate and *n*-butanol fractions actively scavenged DPPH and prevented β-carotene bleaching with IC_50_ values significantly (*p* < 0.05) lower than the positive controls, ascorbic acid and BHT, respectively.

**Table 3.** Antioxidant capacities of crude methanol and aqueous extracts and fractions of AT.

	Crude extract		Fractions					
	Methanol	Aqueous	Hexane	DCM	Ethyl acetate	*n*-Butanol	Water	Controls
AC (μg GAE/mg crude extract/fraction)	0.44 ± 0.02^a^	0.17 ± 0.00^b^	0.38 ± 0.01^a^	0.43 ± 0.01^a^	1.41 ± 0.17^c^	0.93 ± 0.00^e^	0.58 ± 0.05^d^	ND
FRAP (μg TE/mg crude extract/fraction)	1625.63 ± 5.00^a^	580.23 ± 8.23^b^	116.02 ± 0.94^c^	337.42 ± 0.41^d^	1553.10 ± 6.19^a^	1087.39 ± 1.89^f^	794.55 ± 5.75^e^	ND
DPPH (IC_50_ μg/mL)	64.42 ± 5.35^e^	290.60 ± 2.55^a^	271.78 ± 12.56^a^	110.89 ± 7.54^d^	17.07 ± 3.40^b^	13.30 ± 0.78^c^	16.32 ± 2.38^b^	189.91 ± 6.18^f^
β-Carotene (IC_50_ μg/mL)	19.49 ± 3.69^d^	31.55 ± 2.14^a^	38.03 ± 3.76^a^	10.50 ± 0.15^e^	3.46 ± 0.26^b^	4.60 ± 0.63^b^	1.32 ± 0.05^c^	23.92 ± 1.99^d^

Values are expressed as mean ± standard deviation of triplicate determinations. Values in the same row having different superscripts differ significantly (*p* < 0.05). ND: Not determine.

Table 4 shows the phytochemical determination of the crude extracts and fractions of AT. It was observed that the crude methanol extract contained higher amount of phenol, flavonoid, tannin and anthocyanin. Phytochemical determinations of fractions obtained from solvent partitioning of the crude methanol extract were also investigated. It was found that the ethyl acetate and *n*-butanol fractions possessed higher phenol, flavonoid and anthocyanin contents.

**Table 4.** Phytochemical determination of crude methanolic and aqueous extracts and fractions of AT.

	Crude extract		Fractions				
	Methanol	Aqueous	Hexane	DCM	Ethyl acetate	*n*-Butanol	Water
TPC (μg GAE/mg crude extract/fraction)	675.53 ± 0.57^a^	438.48 ± 0.13^b^	19.35 ± 0.12^c^	43.05 ± 0.73^d^	211.74 ± 2.26^e^	293.13 ± 3.45^f^	112.13 ± 0.99^g^
TFC (μg RE/mg crude extract/fraction)	223.11 ± 4.12^a^	170.91 ± 0.73^b^	22.57 ± 1.21^c^	45.30 ± 4.28^d^	272.93 ± 2.51^e^	174.64 ± 3.17^f^	186.79 ± 0.98^g^
TTC (μg catechin/mg crude extract/fraction)	1749.33 ± 33.31^a^	922.67 ± 28.10^b^	30.97 ± 0.00^c^	40.00 ± 7.33^d^	313.55 ± 3.35^e^	210.32 ± 6.80^f^	169.03 ± 9.93^g^
TAC (mg cyanidin-3-glucoside/mL)	13.96 ± 1.95^a^	12.01 ± 1.93^a^	–	–	3.90 ± 0.10^b^	11.05 ± 1.46^c^	6.36 ± 0.52^d^

Values are expressed as mean ± standard deviation of triplicate determinations. Values in the same row having different superscripts differ significantly (*p* < 0.05). – not present.

## Discussion

Medicinal plants have provided the basis for the development of several of our today’s drugs and still many of their therapeutic utilities have not been fully explored (Pan et al. 2013). AT is a medicinal plant which has been extensively used in the Mauritian Traditional Medicinal system to manage various diseases including diabetes and related complications (Mootoosamy & Mahomoodally 2014). However, there is a dearth of scientific data describing the possible inhibitory activities of AT on key enzymes linked to diabetes mellitus and obesity. Diabetes mellitus is a complex metabolic disorder which is closely linked to obesity. Interestingly, α-amylase, α-glucosidase and pancreatic lipase play pivotal role in metabolic functioning and are important pharmacological targets in the management of these complications. To this effect, the present study endeavours to study the inhibitory activities of AT against α-amylase, α-glucosidase, and pancreatic lipase along with its antioxidant potential.

The reduction of postprandial glycaemic level is the front line control in the management of diabetes mellitus. α-Amylase and α-glucosidase are two key enzymes targeted in glycaemic control. The inhibition of these carbohydrate hydrolyzing enzymes cause a prolong carbohydrate digestion and thus blunt postprandial glycaemic rise (Liu et al. 2013). Obesity is closely related to diabetes mellitus and recently the WHO (2014) has reported that 44% of obese suffered from diabetes mellitus worldwide. Furthermore, this corroborates with the hypothesis of Ramirez et al. (2012) suggesting that chronic postprandial glucose and fatty acids rise might contribute to β-cell failure. Pancreatic lipase, a digestive enzyme, has been an important target in the management of obesity (Birari & Bhutani 2007). Indeed, the digestion of fat to glyceride and free fatty acids by pancreatic lipase is a pre-requisite to its uptake. Thus, the inhibition of pancreatic lipase is crucial in the determination of the efficacy of anti-obesity agents (Bustanji et al. 2011). Findings from the present study showed that AT crude methanol extract suppressed α-amylase, α-glucosidase, and pancreatic lipase. Interestingly, the crude methanol extract showed a low inhibitory potential on α-amylase and stronger action on α-glucosidase. Previous reports have indicated that excessive inhibition of α-amylase could result in bacterial fermentation of undigested carbohydrate in the colon and eventually abdominal distention, flatulence and diarrhoea (Dalar & Konczak 2013; Phan et al. 2013). Thus, stronger inhibition of α-glucosidase activity and mild inhibition of α-amylase activity could address the major drawback of currently used hypo-glycaemic agents (Oboh et al. 2012; Kazeem et al. 2013). Fractionation of the crude methanol extract demonstrated that the ethyl acetate and *n*-butanol fractions were the most potent fractions as depicted by their low IC_50_ values. The ethyl acetate/*n-*butanol mixture was observed to possess a higher degree of inhibition against α-amylase and pancreatic lipase. It could be argued that the phytochemicals present in the fractions act on the enzymes in the same or different manner to exert an enhanced inhibition in a synergistic way (Yang et al. 2014), thereby decreasing the concentration of bioactive constituents required to exert inhibition. Interestingly, data from the present study demonstrated that AT was a potent inhibitor of enzymes directly linked to diabetes mellitus and obesity.

Kinetic studies on the most active crude extract showed that the crude methanol extract exerted two modes of inhibitions, namely the noncompetitive and uncompetitive mechanisms. Crude methanol extract of AT clearly displayed a noncompetitive type of inhibition on α-amylase and α-glucosidase, and an uncompetitive inhibition on pancreatic lipase. The most active fractions (ethyl acetate and *n*-butanol fractions) were further studied for their mechanisms of inhibition using different concentrations of the samples. It was observed that the ethyl acetate and *n*-butanol fractions of AT noncompetitively inhibited α-amylase and α-glucosidase. This suggested that the active phytochemicals presented in the sample were pure noncompetitive inhibitors of the enzyme. Noncompetitive inhibitors do not compete with the substrates for the enzyme’s binding site, instead they bind elsewhere (allosteric site) and induce conformational changes, making the binding site inaccessible to the substrates (Kazeem & Ashafa 2015). On the other hand, pancreatic lipase was uncompetitively inhibited by the crude methanol extract, ethyl acetate, and *n*-butanol fractions. Uncompetitive inhibitors bind to enzyme-substrate complex forming an enzyme-substrate-inhibitor complex (Bisswanger 2008; Cornish-Bowden 2013). It is argued that the conformational rearrangement of the enzyme protein structure upon interaction with the substrate allows binding of the uncompetitive inhibitor to the enzyme at a site other than the active site (Sauro 2012).

We also observed that the ethyl acetate and *n*-butanol fractions obtained from polar aprotic and protic solvents, respectively, were the most active samples. It can thus be argued that polar phytochemicals had greater inhibitory capacity on the enzymes and this observation is consistent with Ramirez et al. (2012) and Ablat et al. (2014). On the other hand, previous studies have reported the presence of xanthones (Danthu et al. 2010) and saponins (Gopalsamy et al. 1988) in the leaves of AT. Interestingly, these phytochemicals have been reported to possess anti-obesity (Han et al. 2001; Zhao et al. 2005; Liu et al. 2015) and antidiabetic (Miura et al. 2001; Han et al. 2008; Elekofehinti 2015) properties.

Considering that the samples might exert their antioxidant actions through multiple mechanisms, different antioxidant assays were employed. One antioxidant assay was based on the reduction of molybdenum (IV) to molybdenum (V) under acidic conditions (Chaouche et al. 2014). Previous reports tend to show that phytochemicals present in AT extracts are widely distributed in nature and possess strong antioxidant potential (Wojcik et al. 2010; Kumar & Pandey 2013). The FRAP assay which highlight the hydrogen-donating ability through the scavenging of preformed Fe^3+ ^was also used to study the antioxidant potential of the samples (Dalar & Konczak 2014). The DPPH assay which is routinely used for antioxidant screening due to its simplicity and sensitivity (Umamaheswari et al. 2008) was also used in the present study. Crude methanol extract of AT was found to significantly (*p* < 0.05) scavenge DPPH^•^ compared to ascorbic acid. This finding is consistent with Dai and Mumper (2010) who reported the strong reductive capacities of phenols, flavonoids, tannins, and anthocyanins, which were also present in AT extracts. Fractionation of the crude extract showed the significant activities of ethyl acetate and *n*-butanol fractions, which usually contain polar phytochemicals. β-Carotene undergoes rapid discoloration in the absence of antioxidant and was thus employed to assess the potential of the AT to neutralize linoleate free radicals (Gholivand et al. 2014). Based on the present findings, it can thus be argued that phytochemicals present in the crude methanol extract and potent fractions, neutralized linoleate free radicals and prevent β-carotene discoloration. It was noted that ethyl acetate and *n*-butanol fractions of AT contained the highest amount of phenol, flavonoid, tannin and anthocyanin. These phytochemicals carry a wide range of biological activities and their antioxidant capacities have been extensively documented (Sakulnarmrat & Konczak 2012; Xiao et al. 2013; Dalar & Konczak 2014; Homoki et al. 2016).

## Conclusions

This study provided an insight of the inhibitory potential of AT on α-amylase, α-glucosidase, and pancreatic lipase using *in vitro* assays. The crude methanol extract proved to be a potent inhibitor of α-amylase, α-glucosidase, and pancreatic lipase. Furthermore, solvent partitioning of the crude methanol extract showed that ethyl acetate and *n-*butanol fractions were the most potent fractions and contained the highest amount of phytochemicals. These findings corroborated with the potent enzyme inhibitory activities and antioxidant capacities of the fractions. This study supports the use of AT in the management of diabetes and obesity and provides the rationale for the isolation and characterization of the bio-active components from the ethyl acetate and *n*-butanol fractions.
